# Acoustic‐electric trigeminal‐nerve stimulation enhances functional connectivity in patients with disorders of consciousness

**DOI:** 10.1111/cns.14385

**Published:** 2023-07-31

**Authors:** Min Wu, Marta Concolato, Bettina Sorger, Yamei Yu, Xiaoxia Li, Benyan Luo, Lars Riecke

**Affiliations:** ^1^ Department of Cognitive Neuroscience, Faculty of Psychology and Neuroscience Maastricht University Maastricht The Netherlands; ^2^ Department of Developmental Psychology and Socialization University of Padova Padova Italy; ^3^ Department of Neurology and Brain Medical Centre, First Affiliated Hospital, School of Medicine Zhejiang University Hangzhou China

**Keywords:** acoustic stimulation, disorders of consciousness, electric trigeminal‐nerve stimulation, gamma band, resting‐state functional connectivity

## Abstract

**Aim:**

Disruption of functional brain connectivity is thought to underlie disorders of consciousness (DOC) and recovery of impaired connectivity is suggested as an indicator of consciousness restoration. We recently found that rhythmic acoustic‐electric trigeminal‐nerve stimulation (i.e., musical stimulation synchronized to electrical stimulation of the trigeminal nerve) in the gamma band can improve consciousness in patients with DOC. Here, we investigated whether these beneficial stimulation effects are mediated by alterations in functional connectivity.

**Methods:**

Sixty‐three patients with DOC underwent 5 days of gamma, beta, or sham acoustic‐electric trigeminal‐nerve stimulation. Resting‐state electroencephalography was measured before and after the stimulation and functional connectivity was assessed using phase‐lag index (PLI).

**Results:**

We found that gamma stimulation induces an increase in gamma‐band PLI. Further characterization revealed that the enhancing effect is (i) specific to the gamma band (as we observed no comparable change in beta‐band PLI and no effect of beta‐band acoustic‐electric stimulation or sham stimulation), (ii) widely spread across the cortex, and (iii) accompanied by improvements in patients' auditory abilities.

**Conclusion:**

These findings show that gamma acoustic‐electric trigeminal‐nerve stimulation can improve resting‐state functional connectivity in the gamma band, which in turn may be linked to auditory abilities and/or consciousness restoration in DOC patients.

## Graphical abstract

The novel acoustic‐electric trigeminal‐nerve stimulation enhances resting‐state functional connectivity. The enhancing effect is specific to gamma band and widely spread across the cortex.

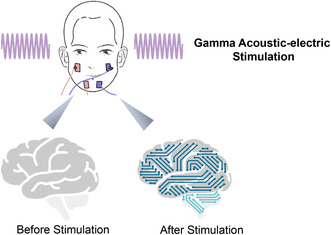

## 1 INTRODUCTION

Even though the mechanisms underlying disorders of consciousness (DOC) remain poorly understood, a growing body of evidence is accumulating regarding the neural correlates of DOC.^1^, ^2^ Some neuroimaging studies have demonstrated abnormalities in the structural and functional connectivity of DOC patients' brains.^3^, ^4^ It has been suggested that a functional disconnection, especially of long‐range corticocortical and thalamocortical connections, is a candidate mechanism underlying impairments of consciousness in DOC patients and that recovery of consciousness is accompanied by partial restoration of these connections.^5^, ^6^ These assumptions are further supported by several resting‐state electroencephalographic (EEG) studies showing alterations in functional connectivity (quantified as synchronization of oscillations between different areas) in various oscillatory frequency bands in DOC patients.^7^, ^8^, ^9^, ^10^ Although the results of these studies are heterogeneous, some suggest that increased level of consciousness or improved DOC‐treatment outcome is associated with decreased functional connectivity in the delta band or increased functional connectivity in other higher frequency bands (i.e., theta, alpha, and beta).^7^, ^9^, ^11^

Thalamocortical gamma oscillations, particularly at 40 Hz, have been proposed as a substrate for consciousness.^12^, ^13^ For example, brain activity in the low gamma range (around 40 Hz) in response to rhythmic auditory stimuli has been suggested as a marker of the integrity of thalamocortical networks in prolonged‐DOC patients.^14^ In fact, activity in this range has been shown to correlate positively with consciousness level in prolonged‐DOC patients^15^, ^16^ and lucid dreaming in healthy subjects.^17^, ^18^ Furthermore, functional connectivity in the high gamma range has been associated with accelerated recovery of consciousness after anesthesia in rats^19^ and with changes in sleep versus wakefulness state in human subjects.^20^ Thus, targeting oscillations and functional connectivity in the gamma band might be a potential therapeutic avenue for DOC.

A common approach to modulate neural oscillations in a specific band involves the non‐invasive application of rhythmic sensory or electrical stimulation.^21^, ^22^ We have recently proposed and validated a novel variant involving the combined application of rhythmic musical stimulation and rhythmic transcutaneous electrical stimulation of the trigeminal nerve (TN) (referred to as “acoustic‐electric TN stimulation”).^23^ In that study, we found that acoustic‐electric TN stimulation in beta (28 Hz) and especially gamma (40 Hz) band has beneficial effects on DOC patients' level of consciousness and neural oscillatory responses at the stimulation frequency. In particular, 40 min of stimulation per day, for five consecutive days, lead to long‐lasting improvements in both the level of consciousness and steady‐state responses to auditory stimulation (ASSRs) at the stimulation frequency.

In fact, each type of gamma stimulation—rhythmic acoustic stimulation and oscillatory electrical stimulation—has previously been shown to entrain gamma oscillations in animals and healthy human individuals^17^, ^24^ and to modulate resting‐state functional connectivity in DOC patients.^25^ Additionally, 40‐Hz trigeminal‐nerve stimulation has been reported to improve consciousness in a DOC patient and this improvement was accompanied by changes in functional connectivity, as measured with functional magnetic resonance imaging (fMRI).^26^ According to the current body of evidence, we hypothesized that (i) gamma acoustic‐electric TN stimulation can improve gamma‐band functional connectivity in DOC patients and (ii) the putative change in resting‐state functional connectivity is the mechanism underlying the beneficial effects of TN stimulation on consciousness level and neural oscillatory responses as found in our previous study.

## 2 METHODS

### 2.1 Patients

Data were analyzed from 63 patients diagnosed with DOC based on Coma Recovery Scale‐Revised (CRS‐R) (21 in an unresponsive wakefulness syndrome, 42 in a minimally conscious state), which were obtained in our previous study.^23^ Initially, a total of 72 patients were recruited and assigned to three groups matched as much as possible for demographic characteristics (i.e., age, etiology, and time since injury) and CRS‐R score at pretest. Sixty‐three out of 72 patients completed the study and were included for the current analysis, resulting in 23 patients in the gamma‐TN stimulation group, 20 patients in the beta group and 20 patients in the sham group. The study was approved by the local research‐ethics committee and registered as a clinical trial on www.clinicaltrials.gov (NCT04435301). Informed consent was obtained from the patients' legal surrogates.

### 2.2 Study design

**FIGURE 1:**
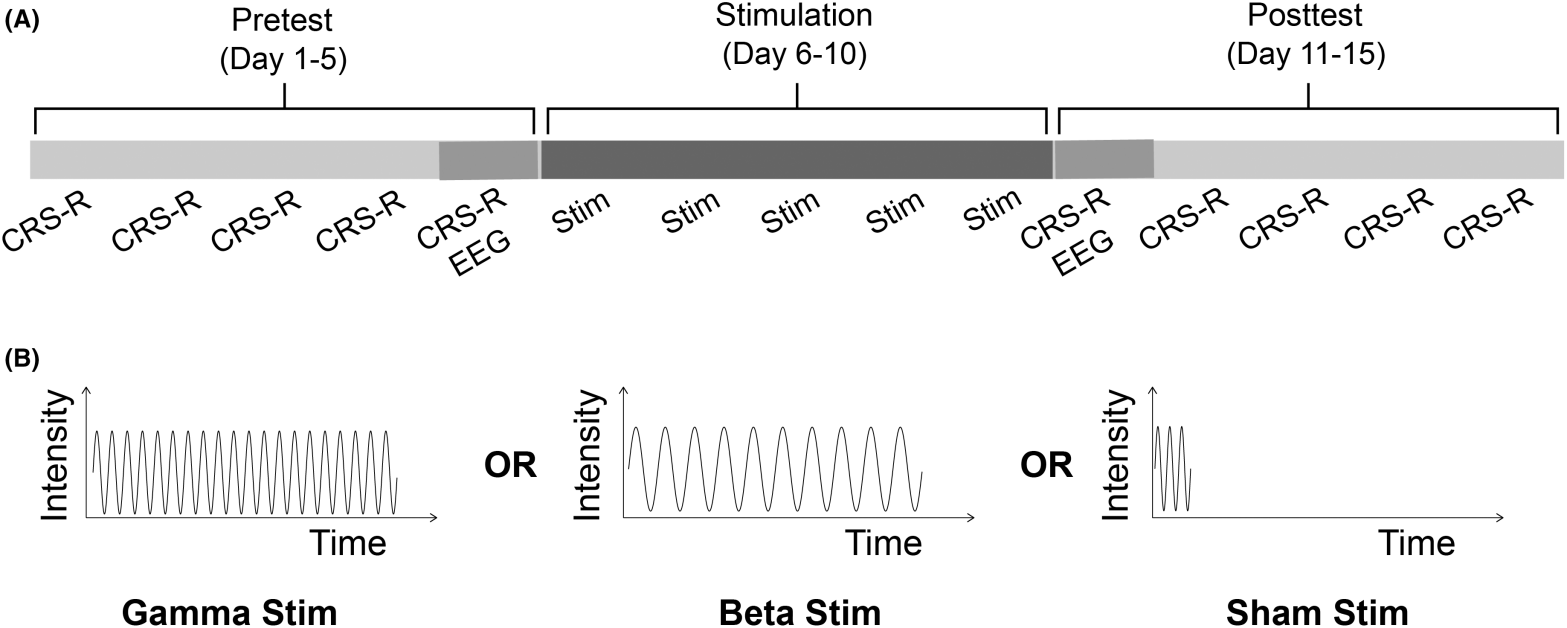
Schematic of experimental procedure. (A) Each patient underwent a 15‐day long experimental procedure consisting of three 5‐day long consecutive phases: pretest, stimulation, and posttest. The pretest and posttest phases involved administration of daily behavioral assessments (CRS‐R) and a single neural assessment (ca. 55 min EEG) on the day immediately before and after the stimulation phase, respectively. (B) The stimulation phase involved daily administration of 40 min of rhythmic gamma (left) or beta (center) acoustic‐electric trigeminal nerve stimulation, or sham (right) stimulation. CRS‐R, Coma Recovery Scale‐Revised; EEG, electroencephalography.

The study followed a mixed 2 × 3 design with the within‐subject factor *time* (pretest and posttest) and the between‐subject factor *stimulation* (gamma, beta, and sham). The study consisted of three consecutive phases of 5 days each: pretest phase, stimulation phase, and posttest phase (Figure 1). Behavioral assessments were administrated during pretest and posttest phases on each of five consecutive days, as repeated CRS‐R assessments have been shown to improve diagnostic accuracy.^27^ A single session of neural assessment (ca. 55 min of continuous EEG recording) was performed on the fifth day during the pretest phase and on the first day of the posttest phase. During the stimulation phase, each group of patients received a single session of gamma, beta, or sham acoustic‐electric TN stimulation on each of the five consecutive days. The beta and sham stimulation were included to further test whether putative stimulation effects on connectivity were specific to the gamma‐stimulation frequency and caused by the actual acoustic‐electric TN stimulation (but not a placebo effect).

### 2.3 Stimulation

The stimulation phase involved administration of rhythmic acoustic‐electric stimulation, composed of simultaneous musical stimulation and TN stimulation.

Acoustic stimulation was a continuous 40 min long musical stream (10 concatenated pieces of music). Amplitude modulations (AM) with 40 or 28 Hz (gamma stimulation and beta stimulation, respectively; sinusoidal waveform, modulation depth: 100%) were applied to the sequence. The onset/offset of each excerpt was ramped up/down using 5 s long ramps. The acoustic stimulation was presented diotically through insert earphones at a fixed sound level (50 dB SPL).

Electric stimulation consisted of non‐invasive application of alternating currents to the patients' face to facilitate rhythmic TN activity. The aforementioned AM and ramping up/down steps were applied analogously to the current waveform. The current intensity was fixed at ±8 mA based on prior tests with healthy volunteers, and related research on TN stimulation.^26^, ^28^ The current was applied using two pairs of square‐shaped rubber electrodes (size: 3 cm × 3 cm) placed at the bilateral middle and lower part of the patient's face to stimulate the second and third branches of the TN (maxillary nerve and mandibular nerve, respectively). The electrodes were adhered to the patients' skin using conductive paste and the impedance was kept below 5 kΩ. The electric stimulation was delivered simultaneously with the acoustic stimulation using a battery‐powered DC stimulator (DCSTIMULATOR MC; NeuroConn) and lasted 40 min.

Sham stimulation was identical to the gamma stimulation above, except that the acoustic and electric stimulation were slowly ramped down after the first 30 s of stimulation. Note that the patient blinding for acoustic stimulation was not optimal, as patients in the sham group might have been able to notice the muting of the stimulation. More detailed description of acoustic‐electric TN stimulation can be found in Wu et al.^23^

### 2.4 Behavioral assessment

Patients' level of consciousness was assessed by two clinicians (J.G. and X.L.) using the CRS‐R during pretest and posttest phases.^29^

### 2.5 Neural assessment

#### 2.5.1 EEG recording

EEG data were collected at the patients' bedside using a 64‐channel active BrainCap (Brain Products GmbH) in the standard 10–20 system. An additional electrooculography electrode was placed at the suborbital ridge of the right eye. The ground electrode was AFz, and all EEG electrodes were referenced to scalp position FCz. Electrode impedances were kept below 10 kΩ. The EEG recordings were online band‐pass‐filtered between 0.01 and 70 Hz, and digitized with a sampling rate of 1 kHz. The EEG recording included two sessions, respectively, at pretest and at posttest. Each session began with a 5‐min resting‐state EEG measurement, immediately followed by approximately 50 min of passive listening to amplitude‐modulated speech. The speech stimuli were repetitive word quartets. Half of the stimuli carried the 40‐Hz AM and the other half carried the 28‐Hz AM. The AM speech served the assessment of the ASSR.^23^

#### 2.5.2 EEG preprocessing

Data preprocessing and analysis were performed offline using EEGLAB 2019.1^30^ and MATLAB 9.4. First, bad channels were identified based on voltage distribution, with kurtosis higher than five as a criterion for rejection. These channels were replaced by interpolating between the surrounding electrodes (spherical spline interpolation; number of interpolated channels: 2.4 ± 2.2, mean ± SD across patients). Second, data were band‐pass‐filtered between 1 and 45 Hz using a Butterworth Infinite Impulse Response (IIR) filter (zero phase shift, filter order: 6). Third, the interpolated channel data were re‐referenced to an average reference. Fourth, independent component analysis was applied to the data using a second‐order blind‐identification algorithm.^31^ Artifactual components were identified and discarded (number of artifactual components: 21.7 ± 8.1; mean ± SD across patients) using the EEGLAB plugin ICLables.^32^ The data were reconstructed based on the remaining components. Next, the artifact‐reduced, continuous data were separately filtered into beta (12–30 Hz) and gamma (30–45 Hz) bands using the same IIR filter as above. Finally, the filtered data were segmented into 4‐s non‐overlapping epochs, resulting in 75 epochs per patient that were used for further analysis.

#### 2.5.3 Functional connectivity

Functional connectivity was assessed using a phase‐lag index (PLI) that quantifies phase synchronization between two time‐varying signals and is considered to be less sensitive to the undesired influence of common sources (volume conduction and/or active reference electrodes) compared with other commonly used measures of connectivity.^33^ The PLI quantifies the degree to which two signals are consistently either in phase advance (phase lead) or in phase delay (phase lag) with each other, and disregards phase lags of 0 modulo π, that are attributed to the influence of common sources. PLI values range from 0 to 1, with 0 representing no phase coupling, and 1 representing perfect phase coupling; the latter can be interpreted as one signal being consistently either in phase advance or in phase delay with respect to the other. The calculation of PLI followed the steps below and was conducted separately for the gamma and beta band: First, within each epoch, the instantaneous phase was determined using the Hilbert transformation. Then, the PLI between two signals for a certain epoch was obtained from a time series of phase differences $∆\phi \left(t_{k}\right),\;k=1\ldots N$, as

(1) $$\mathrm{PLI}=\left|\left\langle \mathrm{sign}\left[\sin∆\phi \left(t_{k}\right)\right]\right\rangle \right|$$

where $∆\phi \left(t_{k}\right)$ indicates the difference between the instantaneous phases of two channels at time point $t_{k}$. Finally, the mean PLI for each pair of channels was calculated as the arithmetic mean of all epochs and then represented as a 63 × 63 symmetric connectivity matrix comprising the PLI values for each pair of EEG channels.

The whole‐scalp PLI was computed as the average of all pairs of channels. To explore the spatial distribution of resting‐state functional connectivity, we additionally computed region‐specific PLI values by first clustering all channels into four regions of interest (ROIs), which were frontal, central, temporal, and parietooccipital scalp areas (Figure S1), and then averaging the PLI values of all pairs of channels within each ROI (e.g., for frontal: F1–F3, F2–F4, and Fz–F1) and between each pair of ROIs (e.g., for fronto‐central: F1–C1, F1–C3, and Fz–Cz). This resulted in 10 groups of region‐specific PLI values: fronto‐frontal, fronto‐central, frontotemporal, fronto‐parietooccipital, centro‐central, centro‐temporal, centro‐parietooccipital, temporo‐temporal, temporo‐parietoccipital, and parietooccipital‐parietooccipital.

Resting‐state functional connectivity was visualized with the BrainNet Viewer toolbox.^34^ Values of the 63 × 63 matrix below 0.085 were set to zero. Next, the PLI values, indicating the strength of connectivity between channels, were plotted as line thickness (Figure 3).

### 2.6 Statistical analyses

To test whether beta and gamma whole‐scalp PLI significantly changed from pretest to posttest within each treatment group, the two‐sided Wilcoxon signed‐rank test was used; this non‐parametric test was used because the PLI data were not normally distributed according to Shapiro–Wilk tests. The same test was also used to test for a gamma‐PLI change from pretest to posttest within each treatment group for each of the 10 region‐specific electrode groups. To test whether the whole‐scalp gamma‐PLI changes in the gamma‐stimulation group were significantly different from the gamma‐PLI changes in the beta and sham‐stimulation groups, the two‐sided Wilcoxon rank‐sum test was used for between‐subject comparisons. Rank correlations between functional connectivity change and the previously reported changes in CRS‐R and gamma and beta ASSR and their significance were assessed using Spearman's correlation coefficient *ρ*. A significance criterion *α* = 0.05 was used. Type‐I error probabilities inflated by multiple comparisons were corrected by controlling the false discovery rate (FDR).^35^ Effect sizes for non‐parametric statistical tests were quantified using the formula: $r=Z/\sqrt{N}$, where *Z* represents the test statistic and *N* is the sample size.^36^

## 3 RESULTS

### 3.1 Positive aftereffect of gamma acoustic‐electric stimulation on resting‐state functional connectivity

**FIGURE 2:**
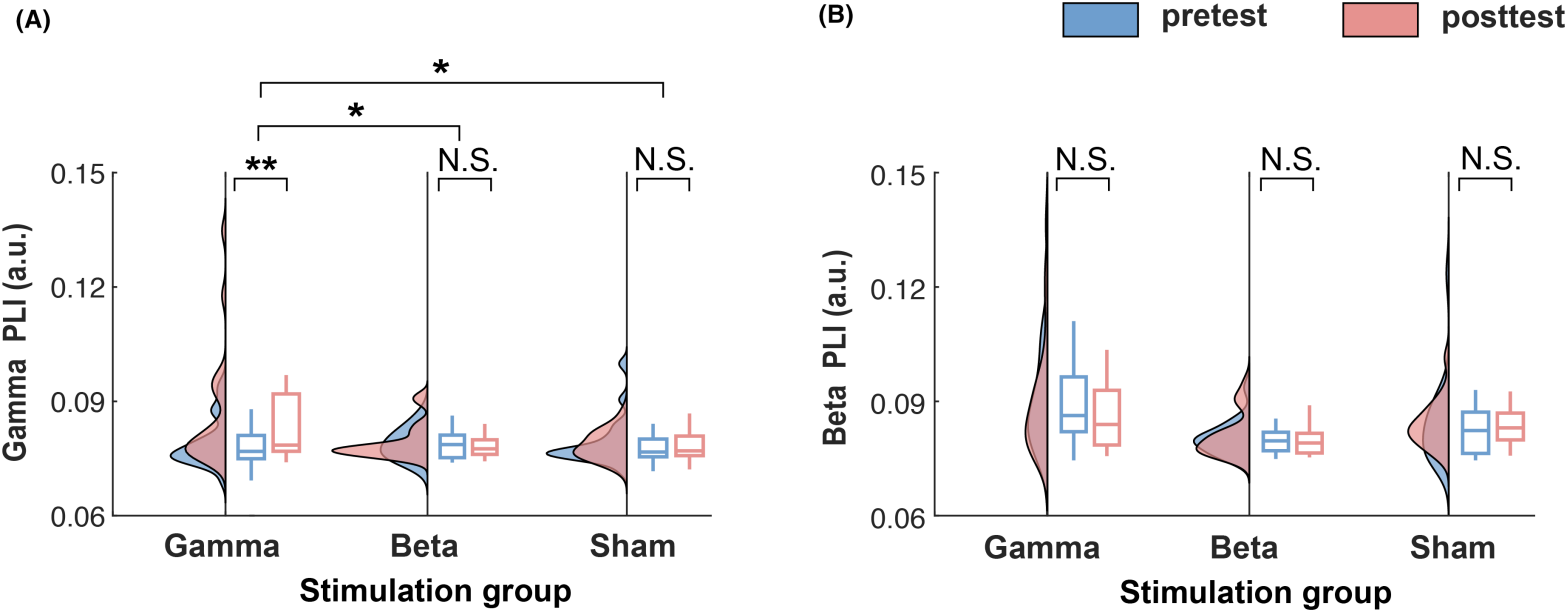
Resting‐state functional connectivity in patients with disorders of consciousness (DOC) in the gamma and beta band before and after rhythmic acoustic‐electric or sham trigeminal nerve stimulation. (A) Whole‐scalp gamma phase‐lag index (PLI) in DOC patients in pretest (blue) and posttest (red). The leftmost pair of plots represents the group of patients who underwent gamma stimulation (gamma‐stimulation group). The middle and rightmost pairs represent the patient group receiving beta stimulation (beta‐stimulation group) and sham stimulation (sham‐stimulation group), respectively. Only gamma stimulation was found to have a strengthening effect on patients' brain connectivity in the gamma band, which was significantly larger than the non‐significant change in the beta and in the sham‐stimulation groups. (B). Same as (A), but for brain connectivity in the beta band. Neither acoustic‐electric nor sham stimulation had an effect on patients' brain connectivity in the beta band. The raincloud plots visualize the data distribution, the horizontal line within each boxplot indicates the median value across participants; the bottom and top edges of the box indicate the first and third quartile values; the whiskers indicate the most extreme values within 1.5 times the interquartile range. N.S., non‐significant, *p < 0.05, **p < 0.01.

To identify whether gamma stimulation modulates resting‐state functional connectivity, we compared the DOC patients' whole‐scalp PLI before versus after gamma stimulation (pretest vs. posttest). We tested this for resting‐state activity in the gamma‐frequency band and a control frequency in the beta band. We found a significant increase in whole‐scalp PLI in gamma band (*Z* = 3.011, *p* = 0.003, *r* = 0.444, Figure 2A, left), but no significant change in beta band (*Z* = 1.947, *p* = 0.052, *r* = 0.287, Figure 2B, left).

**FIGURE 3:**
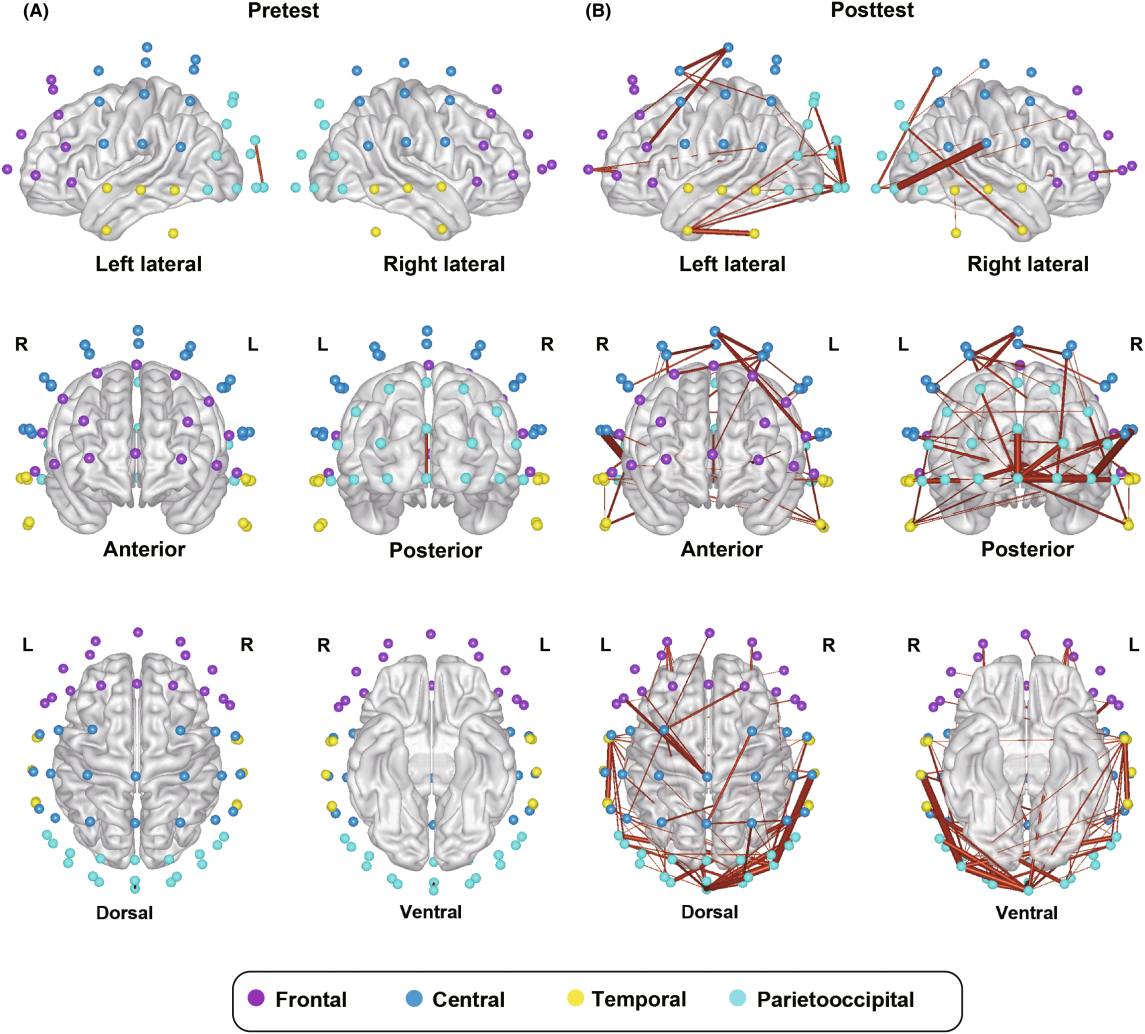
Scalp topography of gamma‐band connectivity in patients with disorders of consciousness (DOC) before and after gamma acoustic‐electric trigeminal nerve stimulation. The spatial distribution of gamma‐band connectivity is depicted in lateral (top row), anterior and posterior (middle row), and dorsal and ventral (bottom row) views of the cerebral cortex. The colored nodes represent the locations of the 63 electroencephalographic electrodes on the scalp. Purple, blue, yellow, and cyan nodes, respectively, represent regions of interest (ROIs) over the frontal, central, temporal, and parietooccipital cortex. The red lines represent gamma‐band connections between pairs of nodes. The line thickness represents the strength of the connection (with thicker lines representing higher phase‐lag index (PLI) values, i.e., stronger connection). A threshold PLI value of 0.085 was applied for clear visualization. (A) Before gamma stimulation, DOC patients were observed to show almost no suprathreshold gamma‐band connectivity within or between any ROIs. (B) After receiving the gamma stimulation, patients showed enhanced gamma‐band connectivity within and between widespread ROIs.

**FIGURE 4:**
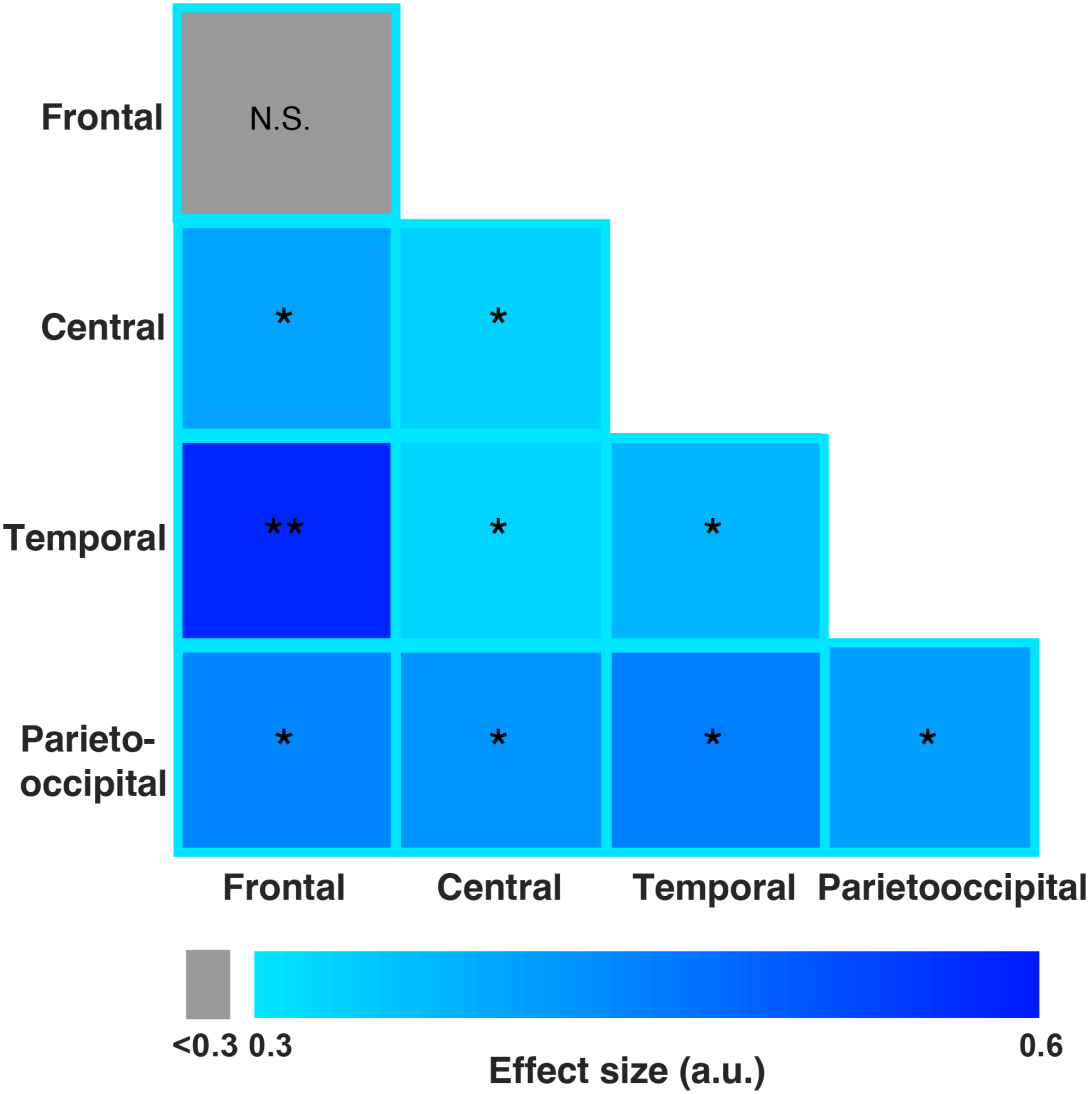
Effect of gamma acoustic‐electric trigeminal nerve stimulation on gamma‐band connectivity in patients with disorders of consciousness (DOC) within and between regions of interest (ROIs). Each cell represents the change in gamma‐band connectivity within a given ROI (cells on the diagonal) or between a given pair of ROIs (cells off the diagonal) after gamma stimulation (pretest vs. posttest). The color of the cell represents the effect size of the change, with darker colors representing stronger phase‐lag index increases. Asterisks represent the statistical significance of the change (N.S., non‐significant, *p < 0.05, **p < 0.01, false discovery rate corrected). Gamma stimulation was found to significantly strengthen gamma‐band connectivity both within ROIs and between each pair of ROIs.

Figure 3 visualizes the scalp topography of patients' resting‐state functional connectivity in the gamma band. It can be seen that the strengthening of brain connectivity from pretest (Figure 3A) to posttest (Figure 3B) involves widespread regions over the scalp. To specify the particular brain regions in which the aftereffect on gamma PLI occurred, we segmented the scalp into four ROIs (Figure S1) and tested the effect of gamma stimulation (pretest vs. posttest) on gamma PLI within each ROI and between each pair of ROIs (see more details in Section 2). As shown by Figure 4, we found a significant increase in PLI both within the majority of ROIs and between all pairs of ROIs, with the largest changes in the frontotemporal region (frontal, central, temporal, parietooccipital ROIs, and their pairings: *Z* > 2, *p* < 0.05, *r* > 0.30; except within frontal ROI: *Z* = 1.916, *p* = 0.055, *r* = 0.282, FDR corrected). Overall, these results suggest that acoustic‐electric gamma‐TN stimulation strengthens gamma‐band connectivity within and between widespread cortical regions.

### 3.2 No aftereffect of beta acoustic‐electric stimulation on resting‐state functional connectivity

To evaluate whether the positive aftereffect of rhythmic acoustic‐electric stimulation on gamma‐band connectivity generalizes from gamma stimulation to other (non‐gamma) stimulation frequencies, we estimated PLI in another patient group receiving the rhythmic acoustic‐electric stimulation at a control frequency in the beta band (i.e., 28 Hz). In contrast to the gamma‐stimulation group, we found this beta‐stimulation group to show no significant change in whole‐scalp PLI in the gamma band (pretest vs. posttest: *Z* = 0.075, *p* = 0.940, *r* = 0.012, Figure 2A, center). Moreover, this observed non‐significant change was significantly smaller than the effect observed in the gamma‐stimulation group (interaction stimulation frequency × time, *Z* = 2.240, *p* = 0.0248, *r* = 0.330, Figure 2A). Similar to the gamma‐stimulation group, the beta‐stimulation group showed no effect on resting‐state functional connectivity in the control frequency band (beta PLI, *Z* = 1.344, *p* = 0.179, *r* = 0.213, Figure 2B, center). The results from the gamma‐ and beta‐stimulation groups together indicate that the observed strengthening of gamma‐band connectivity was induced by the stimulation oscillating at gamma frequency, rather than the stimulation per se.

### 3.3 No aftereffect of sham acoustic‐electric TN stimulation on resting‐state functional connectivity

To further rule out that the strengthening aftereffect of gamma stimulation on gamma‐band connectivity was caused by potential spontaneous recovery and/or a placebo effect, we conducted the experiment in another control group receiving sham stimulation (see Section 2). Similar to the results obtained for the control group (see above), we found this sham‐stimulation group to show no significant change in whole‐scalp PLI from pretest to posttest (gamma PLI: *Z* = 0.261, *p* = 0.794, *r* = 0.041; beta PLI: *Z* = 1.456, *p* = 0.145, *r* = 0.230; Figure 2, right) and the non‐significant gamma‐PLI change was significantly smaller than the effect observed in the gamma‐stimulation group (interaction stimulation type × time: *Z* = 2.118, *p* = 0.034, *r* = 0.312; Figure 2A). Thus, these results support the idea that the observed strengthening of gamma‐band connectivity was induced by the gamma stimulation, rather than spontaneous recovery and/or a placebo effect.

### 3.4 Changes in gamma‐band resting‐state functional connectivity correlate with changes in auditory ability

**FIGURE 5:**
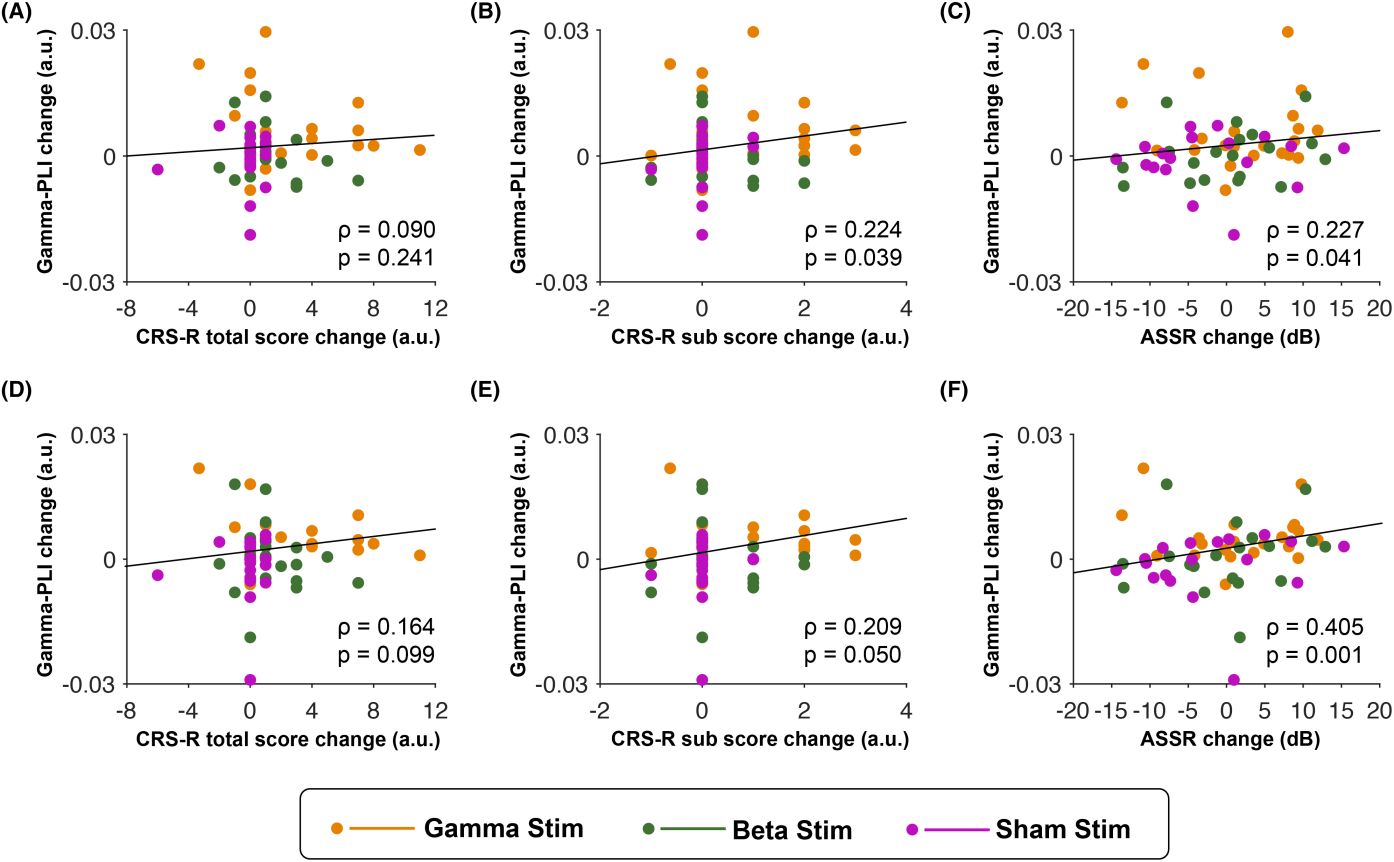
Correlation between changes in gamma‐band resting‐state functional connectivity and changes in the level of consciousness and auditory processing. (A). The scatterplot shows results from a correlation analysis testing for a functional coupling between changes in whole‐scalp gamma‐band connectivity and changes in CRS‐R total score. (B) Same as (A), but for changes in CRS‐R auditory subscore (instead of changes in CRS‐R total score). (C) Same as (A), but for changes in gamma and beta auditory steady‐state response (instead of changes in CRS‐R total score). (D–F) Same results as in (A–C), but after limiting the analysis to the frontotemporal area that showed the largest gamma connectivity change. Correlation coefficient rho (*ρ*) and *p*‐value describe, respectively, the strength and statistical significance of the coupling across all patients. Orange, green, and magenta dots respectively represent data points of individual patients in the gamma‐, beta‐, and sham‐stimulation group. CRS‐R, Coma Recovery Scale‐Revised.

To assess whether the observed changes in brain connectivity were potentially relevant for the patients' improvements in consciousness level as reported in Wu et al.,^23^ we next explored the correlation between patients' changes in whole‐scalp gamma‐band connectivity and their changes in CRS‐R scores (posttest minus pretest). We found no significant correlation between gamma‐band PLI changes and CRS‐R total score changes (*ρ* = 0.090, *p* = 0.241, Figure 5A). Further exploration of the six CRS‐R subscales (auditory function, visual function, motor function, oromotor function, communication ability, and arousal) revealed a significant positive correlation of moderate strength between gamma‐band PLI changes and changes in CRS‐R score at the auditory subscale (*ρ* = 0.224, *p* = 0.039 [uncorrected], Figure 5B). This observation of a putative link between gamma‐band connectivity and auditory ability was further supported by a significant positive correlation between gamma‐band PLI changes and ASSR changes at gamma and beta frequency (i.e., 28 and 40 Hz, *ρ* = 0.227, *p* = 0.041, Figure 5C). Moreover, the changes in gamma‐band connectivity in the frontotemporal region that showed the largest change was also correlated to the changes in CRS‐R auditory subscale and ASSR, but not the changes in CRS‐R total score (CRS‐R total score: *ρ* = 0.164, *p* = 0.099; auditory: *ρ* = 0.209, *p* = 0.050; ASSR: *ρ* = 0.405, *p* = 0.001, Figure 5D–F). Thus, these neural‐behavioral observations suggest that strengthening of gamma‐band connectivity through gamma stimulation can potentially improve auditory processing abilities.

## 4 DISCUSSION

In this study, we found that gamma acoustic‐electric TN stimulation significantly increased gamma‐band resting‐state functional connectivity in widespread areas across the cortex, while it did not affect beta‐band connectivity. We found no such increase in gamma‐band connectivity after beta stimulation or sham stimulation. Additionally, we observed that the gamma‐band PLI changes correlated positively with changes in the ASSR and changes in the CRS‐R auditory subscale, but not to changes in the CRS‐R total score. Overall, these results show that gamma acoustic‐electric TN stimulation can improve gamma‐band resting‐state functional connectivity, beyond potential effects of spontaneous recovery, placebo, or the mere presentation of sensory stimulation per se. They further suggest that the improvement of gamma‐band resting‐state functional connectivity could support auditory abilities in DOC patients.

### 4.1 Rhythmic acoustic‐electric TN stimulation increased resting‐state functional connectivity in DOC patients

TN stimulation has been an effective neuromodulation treatment for attention‐deficit/hyperactivity disorder, migraine, and some other neurological disorders.^37^, ^38^, ^39^ However, its application to DOC patients has remained limited. Fan et al. reported a significant change in degree centrality (a measure of global brain connectivity^40^) in several brain regions (e.g., cerebellum and inferior temporal gyrus) after TN stimulation treatment in a single patient,^26^ thereby providing the first fMRI evidence for the effect of TN stimulation on brain connectivity of a DOC patient. In our previous study,^23^ we found increased ASSR of DOC patients after acoustic‐electric TN stimulation. Our current findings add to these previous findings, which together provide neurophysiological evidence that gamma acoustic‐electric TN stimulation can induce effects on various aspects of cortical activity in DOC patients.

Despite these observations, how exactly gamma acoustic‐electric TN stimulation, as a novel intervention approach for DOC, affects functional brain connectivity remains unclear. A candidate mechanism underlying the effects of acoustic‐electric TN stimulation on resting‐state functional connectivity may relate to the activation of the thalamus and the thalamocortical pathway. A recent study in rats in an unconscious state induced by traumatic brain injury has illustrated that gamma‐TN stimulation activates the lateral hypothalamus and upregulates neuropeptide hypocretin, which are beneficial for promoting consciousness recovery.^41^ Moreover, direct electrical stimulation of the thalamus has been shown to improve consciousness and functional connectivity between the thalamus and the cortex.^42^, ^43^ Similarly, our rhythmic acoustic‐electrical TN stimulation was designed to induce strong synchronous activity in multisensory thalamic nuclei and thus restore connectivity between the thalamus and hierarchically higher structures in the cortex.

### 4.2 Gamma acoustic‐electric TN stimulation increased widespread connectivity

We found that the increase in gamma‐band connectivity after gamma acoustic‐electric TN stimulation is not restricted to a specific region but involves the whole brain. This widespread nature of the effect suggests that gamma stimulation facilitates communication and integration of information across the entire cortex. This notion is supported by a recent study reporting that trigeminal nerve stimulator activates widespread brain areas in mouse models with traumatic brain injury.^44^

### 4.3 The effect of acoustic‐electric TN stimulation on brain connectivity is frequency‐specific

Interestingly, we observed that gamma, but not beta, acoustic‐electric TN stimulation significantly increased brain connectivity, and the effects of gamma stimulation on connectivity were specific to the gamma band and did not generalize to the beta band. These findings highlight that the effects of rhythmic stimulation are frequency‐specific, with a special susceptibility of gamma oscillations for gamma acoustic‐electric stimulation.

External rhythmic stimulation at a given frequency may have the most substantial effect on brain circuits that have a matching resonance frequency. The thalamus has been postulated to regulate arousal via thalamocortical synchronization at 30–40 Hz^45^ and stimulating the central thalamus at 40 Hz has been shown to cause widespread brain activity and arousal.^43^, ^46^ Thus, the frequency specificity of thalamocortical loops suggests that the most effective stimulation frequency is in the gamma band, particularly around 40 Hz. The fact that our scalp EEG results show widespread connectivity changes and gamma‐frequency specificity suggests that these effects involve thalamocortical loops.

### 4.4 Enhanced brain connectivity is related to improved auditory abilities

In contrast with our original hypothesis, we did not find a significant correlation between the change in gamma‐band connectivity and the change in patients' global consciousness state, as measured by the CRS‐R total scores, suggesting the observed global improvement in consciousness is not necessarily accompanied by a gamma‐band connectivity change. Thus, it seems that the gamma‐band connectivity increase is not the mechanism underlying the observed global improvement in consciousness. This interpretation conflicts a previous study by Naro et al.,^47^ which demonstrated a significant correlation between CRS‐R total score and connectivity at gamma frequency in DOC patients.^47^ The difference to the previous results may reflect methodological differences: Naro et al. used a different estimator of functional connectivity (dwPLI, a debiased estimator of the squared weighted PLI), retained only the top 30% of dwPLI values, defined gamma band as 25–40 Hz, and correlated CRS‐R score and dwPLI at a specific point in time, while we used PLI, analyzed all PLI values, defined the gamma band as the range from 30 to 45 Hz, and correlated *changes* between pretest and posttest.

However, we found significant correlations between the gamma‐band connectivity change and changes in the CRS‐R auditory subscore and changes in the ASSR. ASSRs—phase‐locked responses to amplitude‐modulated auditory stimuli—are commonly used as an objective measure of hearing sensitivity.^48^ These results suggest a link between gamma‐band connectivity and auditory processing. Gamma‐band synchronization has been considered as a mechanism for interregional communication that relates to sensory processing and a variety of cognitive processes.^49^, ^50^ For instance, an increase in gamma‐band functional connectivity between auditory cortices and the anterior cingulate cortex was observed during an auditory detection task.^51^, ^52^ In line with these studies, our results suggest that increased gamma connectivity is coupled with improvement of auditory abilities. Moreover, the largest gamma connectivity change, which was observed in frontotemporal regions, also correlated to the changes in CRS‐R auditory subscale or ASSR. Thus, these results suggest that gamma‐band connectivity, particularly in frontotemporal regions, might underlie the observed improvement in auditory processing and auditory abilities.

Although from the correlation results, we cannot conclude that gamma connectivity is the mechanism underlying the consciousness improvement, the observed coupling between the changes in gamma connectivity, and the changes in CRS‐R auditory subscale or ASSR suggests that facilitation of gamma connectivity might underlie the improvement in a specific aspect of consciousness, that is the auditory abilities.

### 4.5 Limitations

Our interpretation requires two cautionary remarks. Firstly, we observed enhanced brain connectivity after gamma acoustic‐electric stimulation. It should be kept in mind that this connectivity was estimated at the level of scalp EEG electrodes, making it challenging to draw firm conclusions about the precise spatial origin of these effects. However, the PLI used here may be less susceptible to the influence of volume conduction than other connectivity measures, and thus, it can still provide valuable insights into connectivity patterns at a coarse, brain‐wide scale. Future research could pinpoint the spatial origin of the observed stimulation effect with methods that offer greater spatial resolution of thalamic and cortical activity, such fMRI. Secondly, our correlation results were not corrected for multiple comparisons; therefore, our interpretation of these results remains tentative and requires further confirmation.

## 5 CONCLUSIONS

In sum, this study illustrates that gamma oscillatory acoustic‐electric TN stimulation can support functional brain connectivity across the cortex and that the observed change in gamma connectivity may play an important role in auditory processing. These findings provide critical insight into the neural effects of the gamma acoustic‐electric TN stimulation approach, which is essential for its clinical translation into a potential novel treatment approach.

## AUTHOR CONTRIBUTIONS

**Min Wu:** Conceptualization; methodology; data curation; formal analysis; writing—original draft; writing—review and editing. **Marta Concolato:** Methodology; writing—original draft; formal analysis; writing—review and editing. **Bettina Sorger:** Conceptualization; supervision; writing—review and editing. **Yanmei Yu** and **Xiaxia Li:** Data collection; writing—review and editing. **Benyan Luo:** Conceptualization; funding acquisition; data curation; writing—review and editing. **Lars Riecke:** Conceptualization; methodology; supervision; writing—original draft; writing—review and editing.

## FUNDING INFORMATION

This work was supported by the National Natural Science Foundation of China (81870817) to B.L. and China Scholarship Council to M.W. (CSC 201906320078).

## CONFLICT OF INTEREST STATEMENT

All authors declare no competing interests.

## Supporting information


Figure S1.

## Data Availability

The data that support the findings of this study are available from the corresponding author upon reasonable request.
