# Supplementary material for: Acoustic‐electric trigeminal‐nerve stimulation enhances functional connectivity in patients with disorders of consciousness
Source: CNS Neurosci Ther. 2023 Jul 31;30(3):e14385. doi: 10.1111/cns.14385 (PMC10928333; doi:10.1111/cns.14385)
Supplement: Supplementary file 1 — Figure S1. [file CNS-30-e14385-s001.docx]

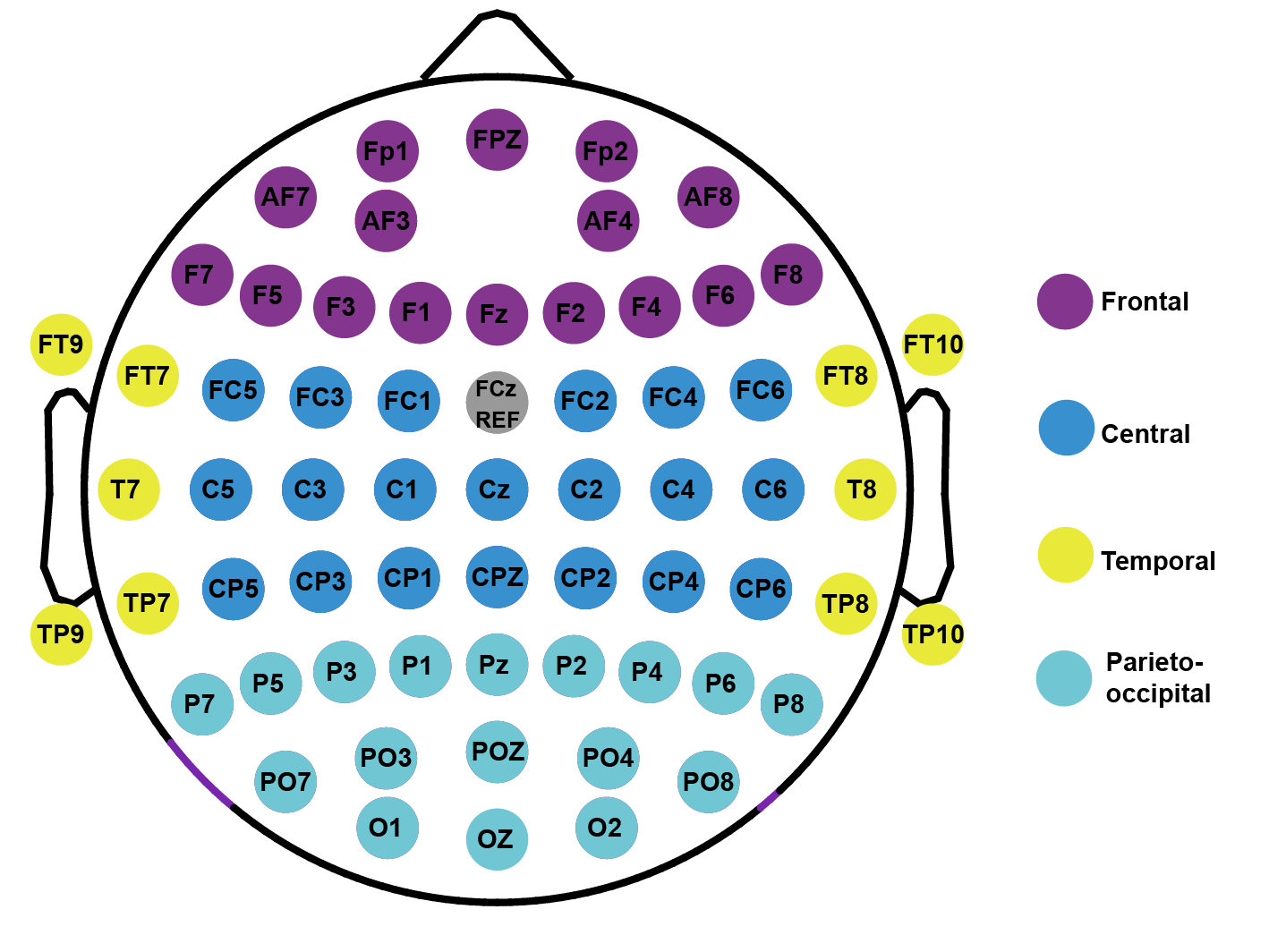


**Figure S1. Segmentation of the scalp into four regions.**

Scalp locations were clustered into four regions of interest: frontal, central, temporal, and parietooccipital regions (represented by the different hues in the figure). Functional connectivity was assessed within each ROI and between each pair of ROIs.
